# MRI-based preoperative WHO grade prediction in meningioma: development and comparison of deep learning, radiomics, clinical, and combined models in multicenter data

**DOI:** 10.1007/s11060-026-05827-6

**Published:** 2026-10-14

**Authors:** Cagin Huryol, Umid Sulaimanov, Murat Yuce, Nafiye Sanlier, Fikret Ozturk, Oyku Ozturk, Irem Altun, Gular Ismayilova, Huseyin Bozkurt, Taha Yasin Altunkaya, Ferdi Afsin, Abdullah Soydan Mahmutoglu, Amine Durmus, Burak Karaaslan, Ilhan Aydin, Mehmet Sabri Gurbuz, Ahmet Tan Cimilli, Okan Turk, Ufuk Erginoglu, Mustafa K. Baskaya

**Affiliations:** 1 Department of Radiology, Gazimagusa State Hospital, Famagusta, Northern Cyprus; 2 Department of Neurological Surgery, School of Medicine and Public Health, University of Wisconsin, CSC K4/822, 600 Highland Avenue, Madison, WI 53792 USA; 3 Icahn School of Medicine at Mount Sinai, Biomedical Engineering and Imaging Institute, New York, NY USA; 4 Department of Neurosurgery, Istanbul Training and Research Hospital, University of Health Sciences, Istanbul, Turkey; 5 Department of Neurosurgery, Bakirkoy Prof. Dr. Mazhar Osman Training and Research Hospital for Psychiatry, Neurology, and Neurosurgery, University of Health Sciences, Istanbul, Turkey; 6 Department of Neurosurgery, Gazi University Faculty of Medicine, Ankara, Turkey; 7 Department of Neurosurgery, Prof. Dr. Suleyman Yalcin City Hospital, Istanbul Medeniyet University, Istanbul, Turkey; 8 Department of Radiology,Istanbul Training and Research Hospital, University of Health Sciences, Istanbul, Turkey; 9 Department of Radiology,Bagcilar Training and Research Hospital, University of Health Sciences, Istanbul, Turkey

**Keywords:** Deep learning, Meningioma, External validation, Prediction, Radiomics, WHO grade

## Abstract

**Purpose:**

The objective of this study was to develop and externally validate complementary magnetic resonance imaging (MRI)-based models and their stacked combinations for preoperative World Health Organization (WHO) grading of intracranial meningioma.

**Methods:**

This retrospective multicenter study included 455 histopathologically confirmed meningiomas: a four-center development cohort (*n* = 346) with leave-one-center-out cross-validation and an external cohort (*n* = 109). Four base models used contrast-enhanced T1-weighted MRI: tumor + peritumoral radiomics, an nnU-Net segmentation-based classifier, frozen DINOv3 transfer-learning features, and clinical variables. All 15 combinations were prespecified and stacked using a linear meta-learner trained on development out-of-fold predictions. Frozen models were externally evaluated without modification.

**Results:**

WHO 2–3 tumors comprised 24.6% overall (112/455; development 20.5%; external 37.6%). External AUCs were 0.732 for nnU-Net, 0.703 for DINOv3, 0.645 for clinical, 0.628 for radiomics. The multimodal ensemble (nnU-Net+DINOv3+Clinical) achieved the highest external AUC (0.742), exceeding radiomics (ΔAUC +0.114, *p* = 0.008) but not nnU-Net (ΔAUC +0.011, *p* = 0.737). Compared with the clinical model, discrimination was higher but nonsignificant at the prespecified threshold (ΔAUC +0.098, *p* = 0.050), while reclassification improved (IDI +0.109, *p* < 0.001). Radiomics-containing combinations performed strongly in development but lost most discrimination externally. The multimodal ensemble had an external calibration slope of 1.32 (Brier 0.190) and net benefit above default strategies at thresholds ≥ 0.23.

**Conclusion:**

The multimodal ensemble achieved the highest external AUC but did not significantly exceed nnU-Net, which was the most transportable single predictor of meningioma grade. Clinical variables did not improve discrimination but enabled interpretable feature-level attribution. Handcrafted radiomics lacked external transportability.

**Supplementary information:**

The online version contains supplementary material available at https://doi.org/10.1007/s11060-026-05827-6.

## Introduction

Meningiomas account for approximately one-third of all primary central nervous system tumors in adults [1, 2]. The 2021 World Health Organization (WHO) classification categorizes meningiomas into Grades 1–3 based on histopathological and molecular features [3]. Compared with Grade 1 tumors, Grade 2–3 meningiomas exhibit more aggressive biological behavior, including increased invasiveness, higher recurrence rates, and poorer clinical outcomes [4–6]. Accurate preoperative prediction of WHO grade may facilitate surgical planning, guide decisions regarding adjuvant radiotherapy, and inform postoperative surveillance strategies [4]. However, definitive grading remains dependent on histopathological examination of the resected tumor [3].

Conventional MRI features associated with high-grade histology often overlap across WHO grades and are subject to substantial interobserver variability [7, 8]. Radiomics provides a quantitative, noninvasive approach for characterizing tumor phenotype and has shown promise for preoperative meningioma grading [9–11]. However, most published radiomics models have been developed and validated within single-center cohorts [7, 12–14], with relatively few undergoing independent external validation to establish true cross-institutional generalizability [15, 16]. Deep learning approaches learn imaging representations directly from MRI and include self-configuring segmentation networks such as nnU-Net [17] and self-supervised foundation models such as the DINO family [18–20]. These approaches may offer greater robustness to inter-center variability. However, direct comparisons with radiomics and clinical models within a unified, leakage-free, externally validated framework remain limited.

We aimed to develop and externally validate complementary MRI-based prediction approaches incorporating handcrafted tumor + peritumoral radiomics, an nnU-Net segmentation-based classifier, DINOv3 transfer-learning features, and clinical variables, and to compare their prespecified stacked combinations for preoperative WHO grade classification of meningiomas. The study was designed with a fully held-out external validation cohort, exact base-model attribution, and prespecified comparative evaluation of the candidate base models and all stacked combinations, in accordance with TRIPOD+AI and CLAIM guidelines [21, 22].

## Materials and methods

### Study design, participants and outcome

**Fig. 1:**
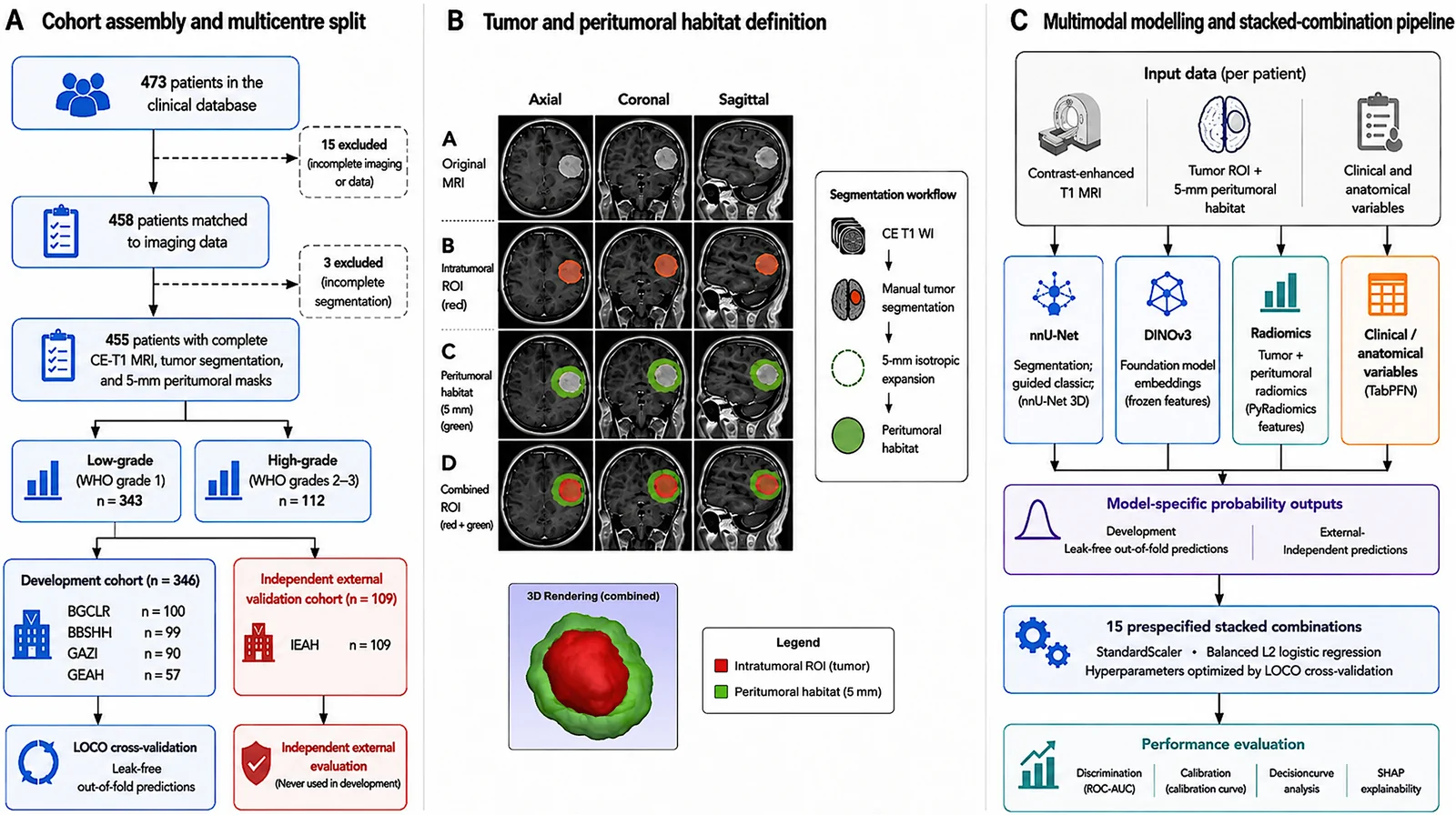
Study design, tumor + peritumoral region definition, and multimodal modeling workflow. (**A**) Cohort assembly and multicentre split. of 473 patients in the clinical database, 15 were excluded because of incomplete imaging or data and 3 because of incomplete segmentation, leaving 455 patients with complete CE-T1 MRI, tumor segmentation, and 5-mm peritumoral region. The final cohort comprised 343 low-grade (WHO grade 1) and 112 high-grade (WHO grades 2–3) meningiomas. Cases were divided into a four-center development cohort (*n* = 346; BGCLR *n* = 100, BRSHH *n* = 99, GAZI *n* = 90, and GEAH *n* = 57), analyzed using leave-one-center-out cross-validation, and an independent external validation cohort (IEAH, *n* = 109). (**B**) Tumor + peritumoral region definition in a representative case. CE-T1 MRI is shown in axial, coronal, and sagittal planes, together with the intratumoral ROI (red), the peritumoral region generated by isotropic 5-mm expansion of the tumor region with subtraction of the original tumor volume and manual removal of non-parenchymal tissue (green), and the combined ROI with 3D rendering. (**C**) Multimodal modeling and stacked-combination pipeline. Four base models were constructed from CE-T1 MRI and clinical/anatomical variables: an nnU-Net segmentation-based classifier, frozen DINOv3 foundation-model embeddings, tumor + peritumoral radiomics, and clinical/anatomical variables (TabPFN). Model-specific probability outputs were generated as development out-of-fold predictions and independent external predictions, then combined into 15 prespecified stacked combinations using a logistic-regression meta-learner optimized by leave-one-center-out cross-validation. Model performance was assessed by discrimination, calibration, decision-curve analysis, and SHAP-based explainability. C*reated in BioRender. Sanlier, N. (2026)* https://BioRender.com/nqrprlw

This multicenter retrospective predictive modeling study included consecutive patients from five tertiary referral centers. All patients had histopathologically confirmed intracranial meningiomas and underwent preoperative contrast-enhanced T1-weighted (CE-T1) MRI. The study aimed to predict WHO meningioma grade preoperatively. The study was approved by the local ethics committee of Istanbul Training and Research Hospital (Date: August 7, 2026; No.: 267). The requirement for informed consent was waived because of the retrospective design. Of 473 patients screened, 458 were matched to imaging data, and the final cohort consisted of 455 patients with complete CE-T1 MRI, tumor segmentation, and corresponding 5-mm peritumoral segmentation (Fig. 1A).

The cohort was partitioned by center into a development cohort comprising four centers (Bagcilar Training and Research Hospital (BGCLR), Bakirkoy Prof. Dr. Mazhar Osman Training and Research Hospital for Psychiatry, Neurology, and Neurosurgery (BRSHH), Gazi University Faculty of Medicine (GAZI), and Prof. Dr. Suleyman Yalcin City Hospital (GEAH); *n* = 346) and an external validation cohort (Istanbul Training and Research Hospital (IEAH), *n* = 109). The external validation cohort was held out throughout all model development and optimization and was used exclusively for comparative external evaluation of the frozen candidate models and stacked combinations. The prediction target was binary WHO grade, classified as low grade (WHO 1) or high grade (WHO 2–3) based on final surgical histopathology, and grade was unavailable to all model components at prediction time. High-grade tumors, defined as positive class, accounted for 20.5% (71/346) of the development cohort and 37.6% (41/109) of the external validation cohort. This difference reflected the natural case mix across participating centers rather than intentional cohort enrichment. Surgery was performed between 2012 and 2025; WHO grade was taken from the original histopathology reports, according to the WHO classification in effect at diagnosis (2007 for tumors operated June 2016: 2016 or 2021 thereafter), without central re-review or routine molecular testing.

### Imaging, segmentation, and preprocessing

Preoperative CE-T1-weighted MRI was acquired on 1.5-T or 3-T scanners from three vendors, with 3D gradient-echo or 2D spin-echo protocols that differed across centers (Supplementary Table S7). No intensity harmonization (e.g., ComBat) was applied; each base model used its own preprocessing: resampling to 1-mm isotropic voxels with z-score normalization for radiomics, per-slice percentile intensity windowing at native resolution for DINOv3, and the default nnU-Net resampling (2 × 1×1 mm) and z-score normalization. Two board-certified radiologists performed manual tumor segmentation in 3D Slicer (version 5.10.0) [23], and a 5-mm peritumoral region was generated automatically around each tumor as an isotropic shell (Euclidean distance in physical space) excluding the tumor itself (Fig. 1B); the shells were then manually edited to remove bone, dura, cerebrospinal fluid, venous sinuses, visible vessels, and air, restricting the peritumoral region to the adjacent brain parenchyma. Both radiologists independently resegmented 23 randomly selected development cases for feature reproducibility analysis. Annotators were blinded to clinical and histopathological data during segmentation.

### Base models

Four base models were developed exclusively within the development cohort to predict the probability of high-grade meningioma (WHO 2–3). Hyperparameter optimization, feature selection, and model calibration were confined to this cohort. The four models are described below.

1. Tumor + peritumoral radiomics. Handcrafted radiomics features (original, Laplacian-of-Gaussian, and wavelet-filtered; 1,223 tumor and 1,209 peritumoral features) were extracted from manually segmented tumor and corresponding 5-mm peritumoral regions using PyRadiomics [24] after resampling to 1-mm isotropic voxels, z-score normalization, and fixed-bin-width discretization (bin width 5). Feature selection was performed within the development cohort only, using reproducibility filtering (ICC > 0.80 from 23 double-read cases), redundancy filtering (|r| >0.90), and TabPFN-based forward selection maximizing the AUC (Supplementary Methods S3), and the selected features were classified with TabPFN [25]. Six final features (three intratumoral and three peritumoral) were retained.
2. DINOv3 transfer-learning features. Imaging features were extracted from tumor-centered axial slices (spanning the tumor’s interquartile craniocaudal range) using a frozen self-supervised DINOv3 Vision Transformer (ViT-B/16) [19]. The extracted features were averaged for each patient and reduced using principal component analysis (PCA). Logistic regression with class balancing was then used for classification, with hyperparameters optimized using leave-one-center-out (LOCO) cross-validation.
3. nnU-Net segmentation-based classifier. An nnU-Net (v2) [17] model was trained to jointly segment the tumor and encode its grade per voxel (label 1, WHO 1; label 2, WHO 2–3) on the CE-T1 volume cropped to the tumor bounding box with a 2-cm margin (Supplementary Methods S2). Six summary statistics of the voxel-wise high-grade probabilities within the tumor region (mean, minimum, maximum, standard deviation, and first and third quartiles) were standardized and combined into a patient-level probability by an L2-regularized logistic regression with class-balanced weights, whose regularization strength was tuned by LOCO cross-validation within the development cohort (Supplementary Methods S1). The five nnU-Net folds were patient-level random splits of the development cohort, each containing patients from all four centers (Supplementary Table S5); development probabilities were out-of-fold estimates, and external probabilities were generated by the five-fold ensemble.
4. Clinical model. A clinical baseline model incorporated age, sex, tumor location, peritumoral edema, bone invasion, venous sinus invasion, and the presence of dural tail [26, 27]. These imaging variables were scored on preoperative CE-T1-weighted MRI by two radiologists in consensus, blinded to histopathological grade. Classification was performed using TabPFN. Postoperative variables, including Ki-67, were excluded to ensure that the model relied exclusively on preoperative information. Missing values (tumor location in two development patients) were median-imputed within each training fold; the external cohort had no missing predictors. As a conventional benchmark, unpenalized logistic regression models using the clinical variables, with and without log-transformed tumor volume from the manual segmentation, were evaluated with the same validation design.

### Ensemble, leakage control, and statistical analysis

The four base models were combined using stacked generalization (Fig. 1C). All 15 model combinations were prespecified before external evaluation. A linear logistic regression meta-learner was trained on the development out-of-fold predictions using LOCO cross-validation, with meta-learner fitting and hyperparameter optimization performed exclusively within the development cohort. The resulting candidate models were then applied to the external cohort. Each of the 15 frozen combinations was evaluated once on the external cohort, and no model was modified, retrained, recalibrated, or discarded after external evaluation; results for all combinations are reported (Supplementary Table S3). Models were compared on both out-of-fold and external validation performance, with discrimination assessed by the area under the receiver operating characteristic curve (AUC).

To prevent leakage, the external validation cohort was excluded from every step of model development, including nnU-Net training, and was used only once to evaluate the frozen models. Within the development cohort, nnU-Net used patient-level five-fold cross-validation, whereas the patient-level classifiers and the meta-learner used LOCO folds, with imputation and scaling fitted within training folds; hyperparameters were selected on the mean LOCO validation AUC, and radiomics feature selection used stratified five-fold splits of the pooled development cohort (Supplementary Methods S2). Development out-of-fold estimates may therefore be optimistic, whereas the external estimates are unaffected. Class imbalance was handled without resampling: the logistic models (DINOv3 and nnU-Net aggregation, meta-learner) used class-balanced weights, and probability balancing was a tuned option of the TabPFN models that was not selected. Performance was also reported separately for each center to assess cross-center variability.

Discrimination was assessed using the AUC with DeLong 95% confidence intervals overall and by center, and the ensemble was compared with individual models using the DeLong test and integrated discrimination improvement (IDI). Classification used a Youden-J threshold derived from development out-of-fold predictions and locked before external validation; alternative operating points targeting 80 and 90% sensitivity were derived post hoc from the development out-of-fold predictions and applied unchanged to the external cohort. Calibration was assessed using the Brier score, calibration-in-the-large, calibration slope, and expected-to-observed ratio, with flexible (LOESS) curves, and also after shifting the intercept to the development prevalence, because class-balanced weights imply a 50% prevalence. Clinical utility was assessed using decision-curve analysis, assuming that a predicted probability above the threshold would prompt planning for potentially aggressive disease; threshold probabilities of 0.15–0.40 were considered clinically plausible. Segmentation quality of nnU-Net was assessed with the Dice coefficient, the 95^th^-percentile Hausdorff distance (HD95), and tumor-volume agreement. Confidence intervals for calibration and IDI were obtained from 2000 bootstrap resamples. Model contributions were evaluated using the linear meta-learner and exact SHAP values of the clinical model (Supplementary Methods S4); these contributions describe the stacked model, not causal or biological effects. Calibration, decision-curve, and explainability analyses were performed for the combination with the highest external AUC. All analyses were performed in Python with a fixed random seed of 42.

## Results

The development cohort comprised patients from BGCLR (*n* = 100), BRSHH (*n* = 99), GAZI (*n* = 90), and GEAH (*n* = 57), whereas the external validation cohort comprised 109 patients from IEAH (Fig. 1A). High-grade tumors accounted for 20.5% of the development cohort and 37.6% of the external validation cohort. Compared with WHO grade 1 tumors, high-grade tumors were significantly associated with male sex, convexity location, peritumoral edema, and bone invasion (all *p* < 0.001; Table 1). Compared with the development cohort, the external cohort had a higher prevalence of high-grade tumors and peritumoral edema (standardized mean difference [SMD] 0.38 for both), less frequent venous sinus contact (overall SMD 0.28) and bone invasion (SMD 0.14), and similar age, sex, tumor location, and dural tail (|SMD| <0.10; Supplementary Table S1).

**Table 1:** Cohort characteristics by WHO grade group (*n* = 455; low = WHO I, high = WHO II-III)

Characteristic	Overall (n = 455)	Low (WHO I) (n = 343)	High (WHO II–III) (n = 112)	*p* value
Age, years, median (IQR)	57.0 (46.0–66.0)	56.0 (46.0–66.0)	59.0 (47.8–68.2)	0.092
Sex, n (%)				**<0.001**
Female	313 (68.8)	251 (73.2)	62 (55.4)	
Male	142 (31.2)	92 (26.8)	50 (44.6)	
Tumor location, n (%)				**<0.001**
Convexity	158 (34.9)	101 (29.6)	57 (50.9)	
Parasagittal/Falx	109 (24.1)	84 (24.6)	25 (22.3)	
Skull base	125 (27.6)	101 (29.6)	24 (21.4)	
Posterior fossa	56 (12.4)	50 (14.7)	6 (5.4)	
Other	5 (1.1)	5 (1.5)	0 (0.0)	
Venous sinus invasion, n (%)				0.751
None	288 (63.3)	219 (63.8)	69 (61.6)	
Contact	96 (21.1)	73 (21.3)	23 (20.5)	
Suspected invasion	71 (15.6)	51 (14.9)	20 (17.9)	
Peritumoral edema, n (%)				**<0.001**
Absent	187 (41.1)	166 (48.4)	21 (18.8)	
Present	268 (58.9)	177 (51.6)	91 (81.2)	
Bone invasion, n (%)				**<0.001**
Absent	379 (83.3)	298 (86.9)	81 (72.3)	
Present	76 (16.7)	45 (13.1)	31 (27.7)	
Dural tail, n (%)				0.049
Absent	334 (73.4)	260 (75.8)	74 (66.1)	
Present	121 (26.6)	83 (24.2)	38 (33.9)	
Ki-67, %, median (IQR)	4.0 (2.0–6.0)	3.0 (2.0–5.0)	8.0 (5.0–15.0)	**<0.001**

Continuous: median (IQR), Mann-Whitney U; categorical: n (%), chi-square / Fisher. Tumor location was unavailable in 2 patients (both low grade); percentages for this variable are calculated on the non-missing denominator. Ki-67 shown descriptively (postoperative) and was not used as a predictor

External AUCs revealed differences across the four baseline models (Table 2). nnU-Net segmentation-based classifier achieved the highest discrimination (AUC 0.732, 95% CI 0.632–0.832), followed by the DINOv3 transfer-learning model (AUC 0.703, 95% CI 0.600–0.805). In contrast, the tumor + peritumoral radiomics model, despite achieving the highest development out-of-fold AUC (0.800), decreased to an external AUC of 0.628 (95% CI 0.522–0.734). The clinical model achieved an external AUC of 0.645 (95% CI 0.536–0.754). A model-free summary of the nnU-Net output, the mean voxel-wise high-grade probability within the tumor, yielded an external AUC of 0.734 (95% CI 0.635–0.833), similar to the fitted aggregation (Supplementary Table S4).

**Table 2:** Discrimination of the base models and the multimodal ensemble (development OOF and external IEAH)

Model	Dev OOF AUC	External AUC (95% CI)	ΔAUC vs ensemble (95% CI)	P	Sens	Spec
Tumor+peritumoral radiomics	0.800	0.628 (0.522–0.734)	+0.114 (0.029 to 0.200)	0.008	0.27	0.88
Clinical	0.615	0.645 (0.536–0.754)	+0.098 (0.000 to 0.196)	0.050	0.73	0.43
DINOv3	0.725	0.703 (0.600–0.805)	+0.040 (−0.012 to 0.092)	0.134	0.78	0.56
nnU-Net	0.726	0.732 (0.632–0.832)	+0.011 (−0.052 to 0.073)	0.737	0.44	0.88
**Ensemble (nnU-Net+ DINOv3+Clinical)**	**0.752**	**0.742 (0.643–0.842)**	**Reference**	–	**0.41**	**0.91**

Models are ordered by external AUC. ΔAUC = nnU-Net+DINOv3+Clinical minus the corresponding model, with DeLong 95% CI and *p* value. Against the clinical model, the ensemble additionally improved reclassification (IDI +0.109, 95% CI 0.052–0.168 [2000 bootstrap resamples]; *p* < 0.001). Operating metrics at the Youden-J threshold (0.51) locked on development out-of-fold predictions; 95% CI by DeLong. The full set of 15 stacked combinations is given in Supplementary Table S3. The four model including tumor + peritumoral radiomics had a lower external AUC (0.718 vs 0.742),consistent with the limited cross-center transportability of radiomics (Supplementary Table S2).

Against the manual reference, nnU-Net segmentation achieved a median Dice coefficient of 0.961 (IQR 0.934–0.977) in the development cohort and 0.969 (0.914–0.980) in the external cohort, with a median HD95 of 1.1 and 1.0 mm (Supplementary Table S6). Computing the patient-level statistics within the nnU-Net-predicted tumor mask instead of the manual segmentation did not change external discrimination (AUC 0.729 vs 0.732).

The six selected radiomics features were evaluated individually in the external validation cohort. Although all six showed strong discrimination during development (univariate AUC 0.74–0.80, all *p* < 10^− 9^), only two peritumoral features remained significant on external validation (Supplementary Table S2). All six features retained the same direction of association in all five centers, but their effect sizes were attenuated externally (Cliff’s δ 0.48–0.60 in development vs 0.09–0.34 externally), and their distributions differed across centers even among WHO grade 1 tumors (Kruskal–Wallis *p* ≤ 0.008), consistent with center-related distribution shift (Supplementary Table S11, Supplementary Figure S4).

All 15 stacked combinations were evaluated in the development and external validation cohorts (Supplementary Table S3). Combinations containing handcrafted radiomics showed the largest decline in performance on external validation; the four-model combination including radiomics (nnU-Net+DINOv3+Radiomics+Clinical) had an external AUC of 0.718. The multimodal ensemble achieved the highest external AUC (0.742). The nnU-Net+DINOv3 model reached an identical external AUC with higher development out-of-fold discrimination (0.784 vs 0.752). Detailed calibration, decision-curve, and explainability analyses are presented for the combination with the highest external AUC. Of the two combinations with identical external AUC, nnU-Net+DINOv3+Clinical is presented rather than nnU-Net+DINOv3 because its clinical component enables variable-level attribution. Its external AUC did not differ significantly from that of the strongest individual model, nnU-Net (0.742 vs 0.732; ΔAUC = 0.011, DeLong *p* = 0.737). Within the meta-learner, nnU-Net contributed most to the final prediction, followed by DINOv3 and the clinical model.

Conventional logistic regression benchmarks achieved external AUCs of 0.683 (95% CI 0.575–0.792) with age, sex, peritumoral edema, bone invasion, and location, and 0.697 (95% CI 0.591–0.802) after adding tumor volume; neither differed significantly from nnU-Net (*p* = 0.44 and 0.53) or from the multimodal ensemble (*p* = 0.22 and 0.26). Tumor volume alone achieved 0.625 (95% CI 0.517–0.734), adding volume to nnU-Net did not improve discrimination (0.728), and the nnU-Net probability remained associated with high grade after adjustment for volume in the development cohort (odds ratio per standard deviation 1.83, 95% CI 1.37–2.43). Substituting a logistic clinical model for TabPFN within the ensemble yielded an external AUC of 0.752 (95% CI 0.652–0.851) (Supplementary Tables S12 and S13).

**Fig. 2:**
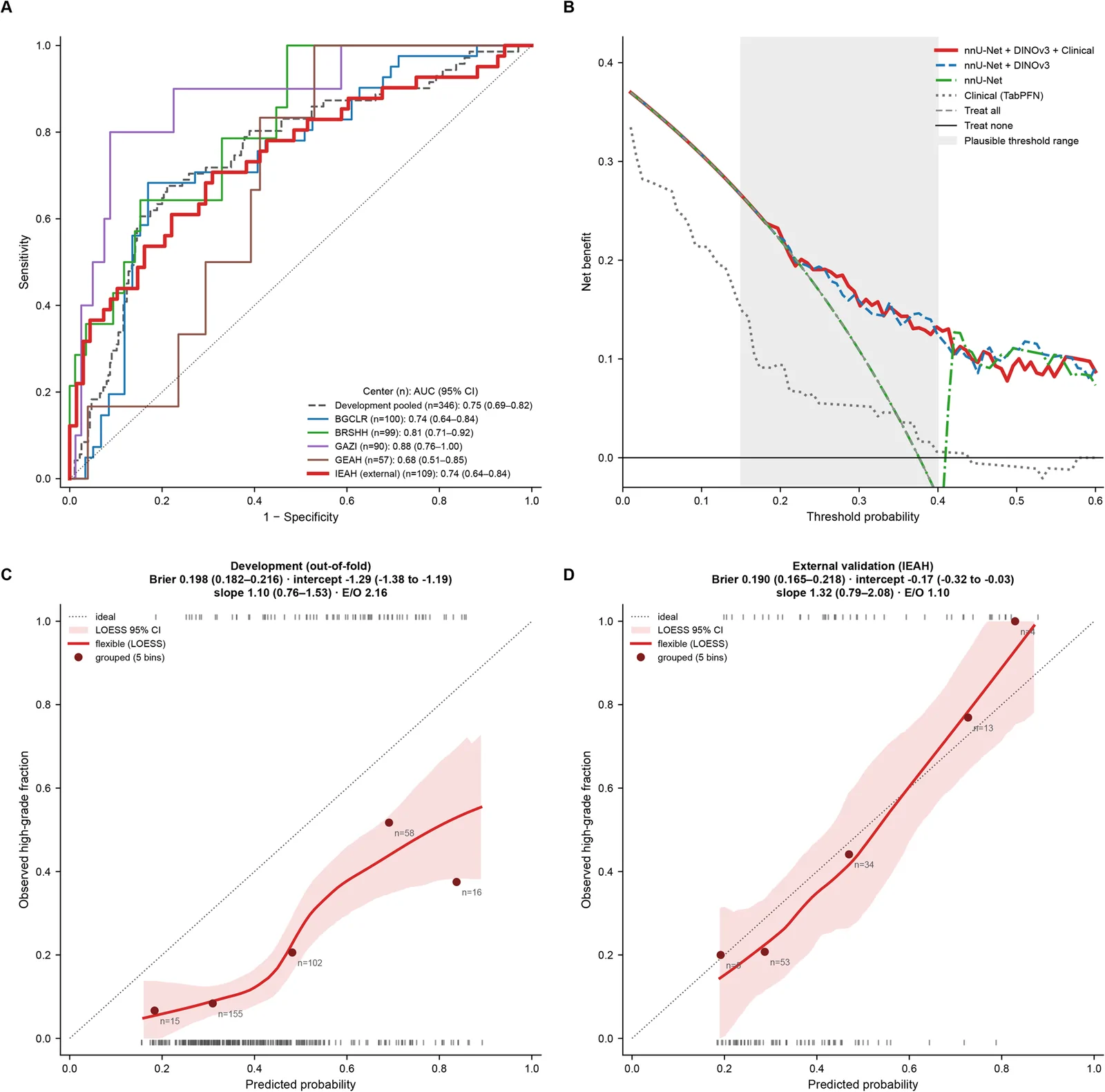
Discrimination, decision-curve analysis, and calibration of the multimodal ensemble. (**A**) center-specific receiver operating characteristic curves for preoperative classification of high-grade (WHO grade 2–3) meningioma. The dashed gray and bold red curves denote the pooled development (*n* = 346) and external validation (IEAH, *n* = 109) cohorts, respectively; AUCs with 95% confidence intervals are shown. (**B**) Decision-curve analysis in the external validation cohort comparing nnU-Net + DINOv3 + clinical with nnU-Net + DINOv3, nnU-Net alone, the clinical model, and treat-all and treat-none strategies; the shaded band marks the clinically plausible threshold range (0.15–0.40). (C–D) calibration in the development cohort (out-of-fold predictions) and in the external validation cohort, respectively: flexible (LOESS) calibration curves with bootstrap 95% confidence bands and observed fractions in five equal-width bins (bin sizes shown); the dotted line denotes ideal calibration. Brier score, calibration-in-the-large, and calibration slope with 95% CIs, and the expected-to-observed ratio are given. Tick marks show predicted probabilities of high-grade (top) and low-grade (bottom) tumors. OOF, out-of-fold

Center-specific AUCs for the multimodal ensemble ranged from 0.683 to 0.880 in development and were 0.742 (95% CI 0.643–0.842) externally (Fig. 2A), with similar discrimination across external scanner vendors and acquisition types (Supplementary Table S7). At the prespecified Youden-J threshold (0.512), external sensitivity and specificity were 0.41 and 0.91 (Supplementary Figure S1); post hoc thresholds fixed on development data for 80 and 90% sensitivity yielded external sensitivities of 0.66 and 0.83, with specificities of 0.71 and 0.49 (Supplementary Table S9). External validation yielded a Brier score of 0.190 (95% CI 0.165–0.218), calibration-in-the-large of −0.17 (−0.32 to −0.03), and calibration slope of 1.32 (0.79–2.08) (Fig. 2C–D); development predictions overestimated risk (calibration-in-the-large −1.29) because of class-balanced weighting, which an intercept shift corrected in development but not externally (Supplementary Table S8, Supplementary Figure S3). In decision-curve analysis, the ensemble and nnU-Net+DINOv3 exceeded both default strategies at threshold ≥ 0.23, nnU-Net alone equaled treat-all up to 0.41, and the clinical model exceeded them only between 0.35 and 0.44 (Fig. 2B, Supplementary Table S10).Compared with the clinical model, the ensemble had a numerically higher AUC (ΔAUC +0.098, *p* = 0.050) and improved reclassification (IDI +0.109, *p* < 0.001); it also exceeded radiomics (ΔAUC +0.114, *p* = 0.008), whereas differences versus DINOv3 (*p* = 0.134) and nnU-Net (*p* = 0.737) were not significant.

**Fig. 3:**
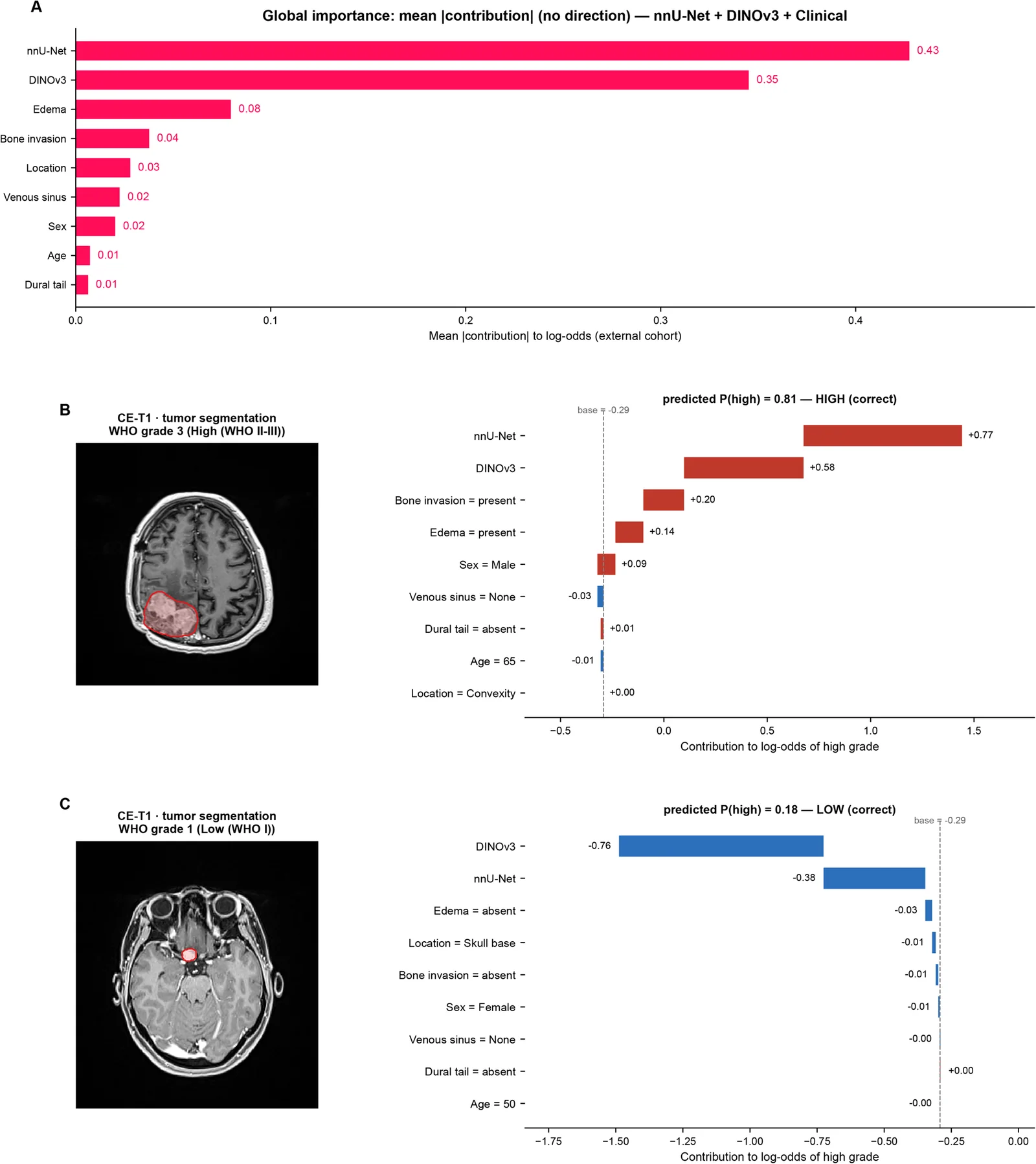
Global and case-level explainability of the multimodal ensemble. (**A**) Global SHAP importance in the external cohort, ranked by mean absolute contribution to the log-odds of high grade. nnU-Net and DINOv3 showed the largest contributions (0.43 and 0.35, respectively), followed by peritumoral edema (0.08), bone invasion (0.04), tumor location (0.03), venous sinus invasion (0.02), sex (0.02), age (0.01), and dural tail (0.01); values are unsigned mean absolute contributions. (**B**) Case-level SHAP explanation for a correctly classified WHO grade 3 meningioma (predicted P[high]=0.81). The high-grade prediction was driven predominantly by concordant contributions from nnU-Net (+0.77 log-odds) and DINOv3 (+0.58), with smaller positive contributions from bone invasion (+0.20) and peritumoral edema (+0.14). (**C**) Case-level SHAP explanation for a correctly classified WHO grade 1 meningioma (predicted P[high] = 0.18). The low-grade prediction was driven predominantly by negative contributions from DINOv3 (−0.76) and nnU-Net (−0.38), with smaller contributions from clinical and anatomical variables. Cases B and C are, respectively, the external WHO grade 3 tumor with the highest and the WHO grade 1 tumor with the lowest predicted probability; the clinical model’s contribution is divided among its variables with exact SHAP values

Because the meta-learner is linear, each prediction decomposes exactly into additive contributions, and the clinical contribution was further divided among its variables using exact SHAP values; because the base-model outputs are correlated (Spearman ρ between nnU-Net and DINOv3 0.41 in development and 0.71 externally), these contributions describe the stacked model rather than causal or biological effects. nnU-Net contributed most, followed by DINOv3, whereas the clinical variables, mainly peritumoral edema, bone invasion, and tumor location, contributed less (Fig. 3A, Supplementary Figure S2). Figure 3B-C shows the external WHO grade 3 tumor with the highest and the WHO grade 1 tumor with the lowest predicted probability; representative cases from each cell of the external confusion matrix, including misclassified cases, are shown in Supplementary Figure S5.

## Discussion

In this multicenter study, nnU-Net was the strongest individual predictor, followed by DINOv3, whereas handcrafted radiomics showed the largest decline from development (AUC 0.800) to external validation (0.628); a similar decline has been reported for radiomics-based Ki-67 prediction [28]. Conventional MRI findings overlap across grades [29], motivating radiomics approaches [12, 30], and single-center CE-T1 or ADC radiomics models have reported accuracies of 75–80% [31, 32] and AUCs up to 0.93 [33]. However, few were externally validated [34]; a meta-analysis reported a pooled AUC of 0.91 [16], and another found validation type to be a significant determinant of performance [15]. The external AUC of our ensemble (0.742) is lower than many reported estimates but may better reflect performance in heterogeneous multicenter practice.

Peritumoral imaging is increasingly recognized as a source of biologically relevant information beyond the tumor itself, reflecting tumor-brain interaction and microenvironmental heterogeneity [35, 36]. Previous studies have associated peritumoral edema and tumor-brain interface characteristics with brain invasion, histological grade, and clinical outcome [37–39]. Multimodal models combining radiologic and radiomic features have also improved prediction of tumor grade, recurrence, and survival [40]. In our study, however, only two of the six selected features retained external significance, both peritumoral: NGTDM Busyness, higher in high-grade tumors, and NGTDM Strength, lower. Both describe local neighborhood intensity variation rather than global tumor appearance [41]. These features may capture heterogeneity at the tumor-brain interface, potentially related to disruption of the arachnoid plane, early infiltration, or edema; however, no histological correlate was available to test these possibilities [37, 38]. All three intratumoral features lost significance. These findings suggest that biological relevance does not necessarily ensure cross-center robustness.

The multimodal ensemble outperformed handcrafted radiomics but not nnU-Net, and adding clinical variables did not improve external discrimination: the radiomics-free nnU-Net+DINOv3 stack matched it externally (AUC 0.742) and exceeded it in development (0.784 vs 0.752), indicating that clinical variables added interpretability rather than discrimination. However, the external cohort was not powered to detect small differences in AUC, and the confidence interval of the difference versus nnU-Net (−0.052 to 0.073) does not exclude a modest benefit of multimodal integration. Its linear meta-learner permitted case-level attribution of both base-model and individual clinical-variable contributions. Because grade is learned per voxel, nnU-Net also returns a 3D segmentation, providing a potential route toward a more automated segmentation-and-grading workflow. However, in the present study patient-level grade probabilities were summarized within the manual reference tumor region; therefore, a fully automated localization-to-segmentation-to-grade workflow was not independently validated. Summarizing the probabilities within the nnU-Net-predicted mask yielded the same external discrimination, but the tumor-centered input crop was still derived from the manual segmentation.

The value of combining imaging and clinical variables has been inconsistent across previous studies. Multiparametric radiomics models developed for related endpoints, such as Ki-67 prediction, have shown favorable performance but often lacked geographically independent external validation [42]. Similarly, combining handcrafted radiomic and deep-learning features has not consistently improved performance [43], whereas deep-learning features have outperformed handcrafted features when the two were compared directly [44]. A four-center study also found no significant external benefit from adding clinical and radiological variables to a radiomics model [45]. Similarly, in a multicenter study of postoperative residual meningioma from overlapping centers, clinical variables provided most of the predictive value, whereas radiomic features showed limited stand-alone discrimination and only modest added value on external validation [46]. In our cohort, a conventional logistic regression model with routine clinical and imaging variables performed comparably to the imaging models externally, so the added value of the learned representations over a well-specified clinical model remains to be established in larger cohorts; nnU-Net probabilities were moderately correlated with tumor volume but retained an independent association with high grade.

The relatively low external sensitivity (41%) may partly reflect transfer of a development-derived decision threshold to an external cohort with a different case mix and score distribution, rather than reduced discriminative performance. Because the AUC is independent of the selected decision threshold, alternative operating points may be obtained by adjusting the classification threshold according to the intended clinical application. For example, a threshold fixed on development data to reach 90% sensitivity identified 83% of high-grade tumors in the external cohort, with a specificity of 49%. This distinction is important when interpreting external validation and is consistent with current recommendations for AI model evaluation in neuro-oncology [22, 47]. The external calibration slope (1.32; 95% CI 0.79–2.08) was compatible with a transported risk gradient, and net benefit exceeded both default strategies at threshold probabilities of 0.23 or higher. Calibration-in-the-large, however, depended on outcome prevalence: the class-balanced meta-learner overestimated risk in the development cohort and would underestimate it externally after adjustment to the development prevalence, so local recalibration of the intercept would be required before clinical use.

Beyond histopathological grading, meningioma classification is increasingly shifting toward imaging biomarkers that capture molecular and tumor-habitat characteristics [5, 48]. For example, unsupervised multihabitat clustering of CE-T1 weighted MRI has shown promising performance in an internal test (AUC 0.73) [49]. MRI-based prediction of molecular risk and specific genomic alterations, such as chromosome 1p loss, has also achieved higher accuracy than histological grade prediction in some studies [50]. Future studies integrating multiparametric imaging, molecular classification, and multimodal deep-learning may provide a more direct assessment of the biological behavior of meningiomas than histopathological grade alone.

Several limitations should be acknowledged. First, this was a retrospective study with external validation at a single independent center, limiting broader generalizability across international populations. Second, the analysis relied exclusively on CE-T1 weighted MRI. Third, WHO grade 2 and 3 tumors were combined because of the small number of grade 3 cases (*n* = 6), precluding separate analysis of grade 3 tumors. Additional limitations include incomplete molecular profiling, segmentation performance assessed only within a tumor-centered crop derived from the manual segmentation, untested inference from a coarse localization, and development out-of-fold estimates that were not fully nested and may therefore be optimistic. The imaging variables of the clinical model were scored in consensus, and their interobserver agreement was not assessed. The reference standard may also have varied across pathological eras. The relative ranking of models should be confirmed in additional external cohorts. In addition, the combination presented in detail was identified by its external AUC; a development-only rule (highest development out-of-fold AUC) would have favored a radiomics-containing combination (development AUC 0.819; external AUC 0.685–0.703), further illustrating that development performance did not predict transportability.

Within the broader landscape of AI in neuro-oncology, the present study highlights the promise of learned deep learning imaging representations for achieving robust cross-institutional performance. Our findings also underscore the need for caution when interpreting high single-center performance reported for approaches such as radiomics, which may be particularly susceptible to site-specific noise and spurious correlations that do not generalize reliably across institutions.

In conclusion, learned deep-learning imaging representations, particularly nnU-Net, were more transportable across centers than handcrafted radiomics, which lost most of its discrimination on external validation despite strong development performance. The multimodal ensemble achieved the highest external AUC but did not significantly exceed nnU-Net, and clinical variables added interpretability rather than discrimination. Prospective validation in larger, geographically diverse cohorts is needed before clinical implementation.

## Electronic supplementary material

Below is the link to the electronic supplementary material.

Supplementary Material 1

## Data Availability

No datasets were generated or analysed during the current study.

## Abbreviations

ADC: Apparent diffusion coefficient
AI: Artificial intelligence
AUC: Area under the receiver operating characteristic curve
BGCLR: Bagcilar Training and Research Hospital
BRSHH: Bakirkoy Prof. Dr. Mazhar Osman Training and Research Hospital
CE-T1: Contrast-enhanced T1-weighted
CI: Confidence interval
CLAIM: Checklist for Artificial Intelligence in Medical Imaging
DL: Deep learning
GAZI: Gazi University Faculty of Medicine
GEAH: Prof. Dr. Suleyman Yalcin City Hospital
ICC: Intraclass correlation coefficient
IDI: Integrated discrimination improvement
IEAH: Istanbul Training and Research Hospital
LOCO: Leave-one-center-out
MRI: Magnetic resonance imaging
NGTDM: Neighborhood gray-tone difference matrix
OOF: Out-of-fold
ROC: Receiver operating characteristic
ROI: Region of interest
SHAP: SHapley Additive exPlanations
TabPFN: Tabular prior-data fitted network
TRIPOD+AI: Transparent Reporting of a multivariable prediction model for Individual Prognosis Or Diagnosis + Artificial Intelligence
WHO: World Health Organization

## Declarations

### Submission statement

This manuscript is original and has not been submitted elsewhere in part or in whole.
