# Supplementary material for: MRI-based preoperative WHO grade prediction in meningioma: development and comparison of deep learning, radiomics, clinical, and combined models in multicenter data
Source: J Neurooncol. 2026 Oct 14;180(1):18. doi: 10.1007/s11060-026-05827-6 (PMC13668925; doi:10.1007/s11060-026-05827-6)
Supplement: Supplementary file 1 [file 11060_2026_5827_MOESM1_ESM.docx]

****Supplementary Figure S1.** Youden-J confusion matrices of the multimodal ensemble by development center and independent external validation cohort. At the development-derived threshold (0.512), the external operating point favored specificity (sensitivity 0.41, specificity 0.91), whereas development centers showed more balanced operating points (BGCLR 0.68/0.83, BRSHH 0.64/0.74, GAZI 0.80/0.89, and GEAH 0.50/0.67).**

****Supplementary Figure S2.**** Global SHAP beeswarm plot for the multimodal ensemble in the external cohort. Each point represents one patient, and rows are ordered by mean absolute contribution to the log-odds of high grade. The clinical model’s contribution is divided among its variables with exact SHAP values without rescaling (Supplementary Methods S4).

**Supplementary Figure S3.** Calibration of the multimodal ensemble after an intercept shift to the development prevalence (20.5%) in the development cohort (left, out-of-fold) and the external cohort (right). Flexible (LOESS) curves with bootstrap 95% bands, observed fractions in five equal-width bins, and tick marks of predicted probabilities for high-grade (top) and low-grade (bottom) tumors. The shift corrects calibration-in-the-large in development, whereas risk is underestimated in the external cohort, whose prevalence is higher (37.6%).

**Supplementary Figure S4**. Univariate discrimination of the six selected radiomic features by center (AUC in the development direction with DeLong 95% CI). All features retain the same direction in every center, with the lowest AUC in the external center (IEAH).

Supplementary Figure S5. Representative external cases for each cell of the confusion matrix at the prespecified threshold (0.512): the patient with the median predicted probability among true positives (A, n = 17), false negatives (B, n = 24), false positives (C, n = 6), and true negatives (D, n = 62). Left, CE-T1-weighted slice with the tumor segmentation; right, additive contributions to the log-odds of high grade (base value shown as dashed line).

Supplementary Table S1. Development vs. external (IEAH) cohort comparability, with standardized mean differences

| Characteristic | Overall (n=455) | Development (n=346) | External IEAH (n=109) | p | SMD |
| --- | --- | --- | --- | --- | --- |
| High grade (WHO 2–3), n (%) | 112 (24.6) | 71 (20.5) | 41 (37.6) | <0.001 | 0.38 |
| Age, years, median (IQR) | 57.0 (46.0-66.0) | 58.0 (46.0-66.0) | 54.0 (46.0-65.0) | 0.473 | 0.05 |
| Female, n (%) | 313 (68.8) | 240 (69.4) | 73 (67.0) | 0.637 | 0.05 |
| Male, n (%) | 142 (31.2) | 106 (30.6) | 36 (33.0) |  |  |
| Convexity, n (%) | 158 (34.9) | 120 (34.9) | 38 (34.9) | 0.988 | 0.06 |
| Parasagittal/Falx, n (%) | 109 (24.1) | 83 (24.1) | 26 (23.9) |  |  |
| Skull base, n (%) | 125 (27.6) | 96 (27.9) | 29 (26.6) |  |  |
| Posterior fossa, n (%) | 56 (12.4) | 41 (11.9) | 15 (13.8) |  |  |
| Other, n (%) | 5 (1.1) | 4 (1.2) | 1 (0.9) |  |  |
| Peritumoral edema absent, n (%) | 187 (41.1) | 157 (45.4) | 30 (27.5) | 0.001 | 0.38 |
| Peritumoral edema present, n (%) | 268 (58.9) | 189 (54.6) | 79 (72.5) |  |  |
| Bone invasion absent, n (%) | 379 (83.3) | 284 (82.1) | 95 (87.2) | 0.241 | 0.14 |
| Bone invasion present, n (%) | 76 (16.7) | 62 (17.9) | 14 (12.8) |  |  |
| Venous sinus - none, n (%) | 288 (63.3) | 212 (61.3) | 76 (69.7) | 0.053 | 0.28 |
| Venous sinus - contact, n (%) | 96 (21.1) | 82 (23.7) | 14 (12.8) |  |  |
| Venous sinus - suspected invasion, n (%) | 71 (15.6) | 52 (15.0) | 19 (17.4) |  |  |
| Dural tail absent, n (%) | 334 (73.4) | 252 (72.8) | 82 (75.2) | 0.709 | 0.05 |
| Dural tail present, n (%) | 121 (26.6) | 94 (27.2) | 27 (24.8) |  |  |

*Continuous: median (IQR), Mann-Whitney U; categorical: n (%), chi-square/Fisher. SMD, absolute standardized mean difference between the external and development cohorts (for variables with more than two categories, the multi-category SMD of Yang and Dalton); |SMD| > 0.10 was considered a meaningful imbalance. The external cohort had a higher prevalence of high-grade tumors and peritumoral edema, less frequent venous sinus contact and bone invasion, and similar age, sex, tumor location, and dural tail.*

Supplementary Table S2. Selected tumor + peritumoral radiomic features: univariate discrimination in development and external cohorts

| Feature | Dir. | Dev. AUC | Dev. p | External AUC | External p |
| --- | --- | --- | --- | --- | --- |
| Tumor - NGTDM Strength (original) | − | 0.773 | 1.3e-12 | 0.596 | 9.4e-02 |
| Tumor - GLCM IMC2 (wavelet-HHL) | − | 0.740 | 4.4e-10 | 0.564 | 2.6e-01 |
| Tumor - GLCM IMC1 (wavelet-LHL) | + | 0.768 | 3.5e-12 | 0.576 | 1.9e-01 |
| Peritumoral - NGTDM Busyness (wavelet-LLL) | + | 0.798 | 8.8e-15 | 0.642 | 1.3e-02 |
| Peritumoral - NGTDM Strength (wavelet-LHL) | − | 0.747 | 1.3e-10 | 0.670 | 3.0e-03 |
| Peritumoral - GLCM IMC2 (wavelet-LLL) | − | 0.761 | 1.2e-11 | 0.547 | 4.2e-01 |

*Univariate ROC-AUC (oriented ≥0.5; Dir. = direction of association with high grade) and Mann-Whitney U p. Features were selected on development folds only; external columns are a held-out check, not used in selection. Only the two peritumoral neighborhood-texture features (NGTDM Busyness, NGTDM Strength) remained statistically significant externally, illustrating the limited cross-center transportability of handcrafted radiomics features.*

Supplementary Table S3. All 15 stacked combinations of the four base models, ranked by external validation AUC

| Rank | Model combination | Dev. OOF AUC | External AUC (95% CI) | Sens. | Spec. | F1 |
| --- | --- | --- | --- | --- | --- | --- |
| 1 | nnU-Net + DINOv3 + Clinical | 0.752 | 0.742 (0.643-0.842) | 0.41 | 0.91 | 0.53 |
| 2 | nnU-Net + DINOv3 | 0.784 | 0.742 (0.643-0.842) | 0.41 | 0.94 | 0.55 |
| 3 | nnU-Net + Clinical | 0.700 | 0.739 (0.638-0.839) | 0.39 | 0.88 | 0.49 |
| 4 | nnU-Net | 0.726 | 0.732 (0.632-0.832) | 0.44 | 0.88 | 0.54 |
| 5 | nnU-Net + DINOv3 + Radiomics + Clinical | 0.791 | 0.718 (0.616-0.820) | 0.41 | 0.90 | 0.52 |
| 6 | DINOv3 + Clinical | 0.665 | 0.706 (0.604-0.808) | 0.76 | 0.59 | 0.62 |
| 7 | nnU-Net + DINOv3 + Radiomics | 0.819 | 0.703 (0.600-0.807) | 0.29 | 0.94 | 0.42 |
| 8 | DINOv3 | 0.725 | 0.703 (0.600-0.805) | 0.78 | 0.56 | 0.62 |
| 9 | nnU-Net + Radiomics + Clinical | 0.789 | 0.699 (0.596-0.802) | 0.41 | 0.90 | 0.52 |
| 10 | DINOv3 + Radiomics + Clinical | 0.761 | 0.689 (0.586-0.792) | 0.41 | 0.78 | 0.47 |
| 11 | nnU-Net + Radiomics | 0.819 | 0.685 (0.580-0.790) | 0.37 | 0.96 | 0.51 |
| 12 | DINOv3 + Radiomics | 0.795 | 0.673 (0.569-0.778) | 0.51 | 0.71 | 0.51 |
| 13 | Radiomics + Clinical | 0.758 | 0.648 (0.542-0.754) | 0.27 | 0.88 | 0.37 |
| 14 | Clinical | 0.615 | 0.645 (0.536-0.754) | 0.73 | 0.43 | 0.55 |
| 15 | Tumor + peritumoral radiomics | 0.800 | 0.628 (0.522-0.734) | 0.27 | 0.88 | 0.37 |

*Stacked models were built on base-model out-of-fold probabilities using StandardScaler and class-balanced, L2-regularized logistic regression, with performance reported at the development-cohort Youden-J threshold. All 15 prespecified combinations were evaluated once on the external cohort; no combination was modified, retrained, recalibrated, or discarded after external evaluation. Rank 1 (nnU-Net+DINOv3+Clinical) and rank 2 (nnU-Net+DINOv3) achieved an identical external AUCs; rank 1 is presented in detail because its clinical component enables variable-level attribution.*

Supplementary Table S4. Patient-level aggregation of the nnU-Net output: standardized coefficients of the aggregation model and discrimination of each statistic alone.

| Statistic of voxel-wise high-grade probability | Standardized coefficient | Development OOF AUC (single statistic) | External AUC (single statistic), 95% CI |
| --- | --- | --- | --- |
| Mean | +0.082 | 0.780 | 0.734 (0.635–0.833) |
| Minimum | +0.010 | 0.694 | 0.611 (0.503–0.719) |
| Maximum | +0.093 | 0.782 | 0.731 (0.630–0.831) |
| Standard deviation | +0.086 | 0.774 | 0.731 (0.631–0.832) |
| First quartile | +0.073 | 0.788 | 0.725 (0.625–0.824) |
| Third quartile | +0.086 | 0.782 | 0.737 (0.639–0.836) |
| Logistic combination of all six (model used) | intercept -0.113 | 0.726 | 0.732 (0.632–0.832) |

*Statistics of the voxel-wise high-grade probability within the tumor region. Coefficients of the L2-regularized, class-balanced logistic regression on standardized statistics (C tuned by LOCO). Single-statistic AUCs use the statistic directly as a score (no fitting); external AUC with DeLong 95% CI.*

**Supplementary Table S5. Composition of the five nnU-Net cross-validation folds (validation portion) by development center**

| **Fold** | **Training, n** | **Validation, n** | **High grade in validation, n** | **BGCLR** | **BRSHH** | **GAZI** | **GEAH** |
| --- | --- | --- | --- | --- | --- | --- | --- |
| 1 | 276 | 70 | 12 | 22 | 12 | 17 | 19 |
| 2 | 277 | 69 | 14 | 21 | 24 | 14 | 10 |
| 3 | 277 | 69 | 12 | 20 | 24 | 19 | 6 |
| 4 | 277 | 69 | 17 | 20 | 20 | 18 | 11 |
| 5 | 277 | 69 | 16 | 17 | 19 | 22 | 11 |

*Random patient-level splits generated by nnU-Net (splits_final.json); every fold contains patients from all four development centers. No external (IEAH) patient is part of any fold.*

**Supplementary Table S6. nnU-Net segmentation quality against the manual reference (A), grading performance by segmentation quality (B), and grading with the nnU-Net-predicted tumor region (C)**

**A**

| **Cohort / center** | **n** | **Dice, median (IQR)** | **Dice < 0.5, n** | **HD95 mm, median (IQR)** | **Manual volume ml, median** | **Volume ICC** | **Volume bias ml (95% LoA)** |
| --- | --- | --- | --- | --- | --- | --- | --- |
| Development (OOF) | 346 | 0.961 (0.934–0.977) | 3 | 1.1 (0.9–3.4) | 20.8 | 0.992 | -0.8 (-10.3 to 8.7) |
| External IEAH (5-fold ensemble) | 109 | 0.969 (0.914–0.980) | 3 | 1.0 (0.9–5.8) | 26.5 | 0.887 | +2.0 (-36.7 to 40.8) |
| BGCLR | 100 | 0.957 (0.945–0.970) | 0 | 1.4 (1.0–3.6) | 28.3 | 0.997 | -0.8 (-5.4 to 3.9) |
| BRSHH | 99 | 0.962 (0.920–0.979) | 1 | 1.1 (0.8–3.1) | 17.8 | 0.987 | -0.8 (-11.6 to 9.9) |
| GAZI | 90 | 0.958 (0.928–0.978) | 2 | 1.0 (1.0–3.6) | 13.9 | 0.985 | -0.4 (-11.6 to 10.7) |
| GEAH | 57 | 0.971 (0.947–0.984) | 0 | 0.9 (0.5–1.9) | 24.2 | 0.994 | -1.4 (-12.3 to 9.5) |

**B**

| **Cohort** | **Model** | **Subgroup** | **n (high grade)** | **AUC (95% CI)** |
| --- | --- | --- | --- | --- |
| Development (OOF) | nnU-Net | Dice ≥ median (0.96) | 173 (38) | 0.721 (0.617–0.825) |
| Development (OOF) | nnU-Net | Dice < median (0.96) | 173 (33) | 0.731 (0.621–0.840) |
| Development (OOF) | nnU-Net + DINOv3 + Clinical | Dice ≥ median (0.96) | 173 (38) | 0.737 (0.644–0.829) |
| Development (OOF) | nnU-Net + DINOv3 + Clinical | Dice < median (0.96) | 173 (33) | 0.767 (0.673–0.861) |
| External IEAH | nnU-Net | Dice ≥ median (0.97) | 55 (15) | 0.592 (0.424–0.760) |
| External IEAH | nnU-Net | Dice < median (0.97) | 54 (26) | 0.793 (0.669–0.916) |
| External IEAH | nnU-Net + DINOv3 + Clinical | Dice ≥ median (0.97) | 55 (15) | 0.675 (0.504–0.846) |
| External IEAH | nnU-Net + DINOv3 + Clinical | Dice < median (0.97) | 54 (26) | 0.760 (0.630–0.889) |

**C**

| **Cohort** | **ROI for the six statistics** | **nnU-Net AUC (95% CI)** | **Ensemble AUC (95% CI)** |
| --- | --- | --- | --- |
| Development (OOF maps) | manual ROI (re-inferred maps) | 0.780 (0.718–0.842) [mean statistic] | – |
| Development (OOF maps) | nnU-Net-predicted ROI | 0.779 (0.716–0.842) [mean statistic] | – |
| External IEAH | manual ROI (re-inferred maps) | 0.732 (0.632–0.832) | 0.742 (0.643–0.842) |
| External IEAH | nnU-Net-predicted ROI | 0.729 (0.629–0.828) | 0.741 (0.642–0.840) |

*A: Dice and HD95 of the binary tumor mask (both labels) within the tumor-centered crop; volume agreement as ICC(2,1) and mean bias (predicted minus manual) with 95% limits of agreement. Development values are out-of-fold; external values come from the five-fold ensemble. B: AUC with DeLong 95% CI in patients above and below the cohort median Dice. C: the six probability statistics computed within the manual or the nnU-Net-predicted tumor region and passed through the frozen classifiers; for development patients only the model-free mean statistic is shown, because the fitted classifier would be applied in-sample.*

**Supplementary Table S7. Acquisition characteristics of the analyzed CE-T1-weighted series by center (A) and discrimination within acquisition strata (B)**

**A**

| **Characteristic** | **BGCLR** | **BRSHH** | **GAZI** | **GEAH** | **IEAH** | **Overall** |
| --- | --- | --- | --- | --- | --- | --- |
| Patients, n | 100 | 99 | 90 | 57 | 109 | 455 |
| DICOM header available, n (%) | 80 (80) | 99 (100) | 90 (100) | 57 (100) | 109 (100) | 435 (96) |
| Vendor: Siemens, n | 4 | 27 | 90 | 0 | 61 | 182 |
| Vendor: Philips, n | 69 | 67 | 0 | 0 | 1 | 137 |
| Vendor: GE, n | 2 | 5 | 0 | 57 | 47 | 111 |
| Field strength 1.5 T, n | 80 | 95 | 29 | 57 | 109 | 370 |
| Field strength 3.0 T, n | 0 | 4 | 61 | 0 | 0 | 65 |
| 3D acquisition, n | 72 | 21 | 88 | 13 | 60 | 254 |
| 2D acquisition, n | 3 | 78 | 2 | 44 | 49 | 176 |
| Gradient echo (GR), n | 77 | 21 | 88 | 13 | 73 | 272 |
| Spin echo (SE), n | 3 | 76 | 2 | 44 | 36 | 161 |
| TR, ms | 25.0 (25.0–25.0) | 450.0 (450.0–633.6) | 1750.0 (9.8–2125.0) | 460.0 (380.0–660.0) | 2000.0 (660.0–2440.0) | 600.0 (25.0–1750.0) |
| TE, ms | 4.60 (4.59–4.61) | 9.10 (9.00–15.00) | 2.93 (2.93–4.76) | 20.00 (12.00–20.00) | 4.36 (3.58–9.02) | 4.60 (3.46–9.11) |
| Flip angle, ° | 30 (30–30) | 69 (69–90) | 9 (8–25) | 90 (90–90) | 8 (8–90) | 30 (9–90) |
| DICOM slice thickness, mm | 1.00 (1.00–1.00) | 5.00 (5.00–5.00) | 1.00 (1.00–1.00) | 5.00 (5.00–6.00) | 1.05 (1.00–5.50) | 1.05 (1.00–5.00) |
| Analyzed volume: largest voxel dimension, mm | 1.00 (1.00–1.10) | 6.00 (6.00–6.00) | 1.00 (1.00–1.00) | 6.00 (3.95–7.00) | 1.05 (1.00–7.00) | 1.05 (1.00–6.00) |
| Analyzed volume: smallest voxel dimension, mm | 0.73 (0.38–0.93) | 0.80 (0.45–0.83) | 0.90 (0.49–0.98) | 0.51 (0.50–0.94) | 0.51 (0.43–0.98) | 0.80 (0.47–0.93) |
| Analyzed volume: largest matrix size | 320 (240–576) | 320 (288–512) | 256 (256–512) | 512 (256–512) | 512 (256–512) | 320 (256–512) |

**B**

| **Cohort** | **Stratum** | **n (high)** | **Model** | **AUC (95% CI)** |
| --- | --- | --- | --- | --- |
| External IEAH | Vendor: GE | 47 (17) | nnU-Net + DINOv3 + Clinical | 0.749 (0.602–0.896) |
| External IEAH | Vendor: GE | 47 (17) | nnU-Net | 0.720 (0.571–0.869) |
| External IEAH | Vendor: Siemens | 61 (24) | nnU-Net + DINOv3 + Clinical | 0.734 (0.593–0.875) |
| External IEAH | Vendor: Siemens | 61 (24) | nnU-Net | 0.740 (0.598–0.882) |
| External IEAH | Field strength: 1.5 T | 109 (41) | nnU-Net + DINOv3 + Clinical | 0.742 (0.643–0.842) |
| External IEAH | Field strength: 1.5 T | 109 (41) | nnU-Net | 0.732 (0.632–0.832) |
| External IEAH | Acquisition: 2D | 49 (18) | nnU-Net + DINOv3 + Clinical | 0.780 (0.643–0.916) |
| External IEAH | Acquisition: 2D | 49 (18) | nnU-Net | 0.771 (0.637–0.904) |
| External IEAH | Acquisition: 3D | 60 (23) | nnU-Net + DINOv3 + Clinical | 0.725 (0.579–0.871) |
| External IEAH | Acquisition: 3D | 60 (23) | nnU-Net | 0.707 (0.557–0.857) |
| Development (OOF) | Vendor: GE | 64 (8) | nnU-Net + DINOv3 + Clinical | 0.728 (0.591–0.864) |
| Development (OOF) | Vendor: GE | 64 (8) | nnU-Net | 0.652 (0.473–0.830) |
| Development (OOF) | Vendor: Philips | 136 (36) | nnU-Net + DINOv3 + Clinical | 0.740 (0.639–0.842) |
| Development (OOF) | Vendor: Philips | 136 (36) | nnU-Net | 0.803 (0.715–0.891) |
| Development (OOF) | Vendor: Siemens | 121 (14) | nnU-Net + DINOv3 + Clinical | 0.810 (0.666–0.953) |
| Development (OOF) | Vendor: Siemens | 121 (14) | nnU-Net | 0.699 (0.501–0.897) |
| Development (OOF) | Field strength: 1.5 T | 261 (54) | nnU-Net + DINOv3 + Clinical | 0.734 (0.660–0.808) |
| Development (OOF) | Field strength: 1.5 T | 261 (54) | nnU-Net | 0.739 (0.659–0.820) |
| Development (OOF) | Field strength: 3.0 T | 65 (7) | nnU-Net + DINOv3 + Clinical | 0.897 (0.730–1.000) |
| Development (OOF) | Field strength: 3.0 T | 65 (7) | nnU-Net | 0.837 (0.594–1.000) |
| Development (OOF) | Acquisition: 2D | 127 (17) | nnU-Net + DINOv3 + Clinical | 0.744 (0.637–0.851) |
| Development (OOF) | Acquisition: 2D | 127 (17) | nnU-Net | 0.612 (0.446–0.777) |
| Development (OOF) | Acquisition: 3D | 194 (41) | nnU-Net + DINOv3 + Clinical | 0.762 (0.664–0.860) |
| Development (OOF) | Acquisition: 3D | 194 (41) | nnU-Net | 0.811 (0.728–0.894) |

*A: DICOM-header values (vendor, field strength, acquisition type, sequence, TR, TE, flip angle, slice thickness; median (range) for continuous values) and geometry of the analyzed volumes (all patients). Part of the BGCLR data were exported without DICOM headers. B: AUC with DeLong 95% CI; development values are LOCO out-of-fold predictions.*

**Supplementary Table S8. Calibration with bootstrap 95% confidence intervals, including prevalence (intercept) adjustment**

| **Model** | **Cohort** | **Probabilities** | **Brier (95% CI)** | **Calibration-in-the-large (95% CI)** | **Slope (95% CI)** | **E/O (95% CI)** |
| --- | --- | --- | --- | --- | --- | --- |
| nnU-Net + DINOv3 + Clinical | Development (OOF) | As published (class-balanced meta-learner) | 0.198 (0.182–0.216) | -1.29 (-1.38 to -1.19) | 1.10 (0.76–1.53) | 2.16 (2.07–2.24) |
| nnU-Net + DINOv3 + Clinical | Development (OOF) | Prior-shifted to development prevalence (20.5%) | 0.143 (0.132–0.155) | +0.07 (-0.02 to +0.16) | 1.10 (0.76–1.52) | 0.95 (0.89–1.01) |
| nnU-Net + DINOv3 + Clinical | Development (OOF) | Prior-shifted to external prevalence (37.6%) (illustration only) | 0.160 (0.145–0.175) | -0.78 (-0.87 to -0.69) | 1.10 (0.76–1.51) | 1.65 (1.57–1.73) |
| nnU-Net + DINOv3 + Clinical | External IEAH | As published (class-balanced meta-learner) | 0.190 (0.165–0.218) | -0.17 (-0.32 to -0.03) | 1.32 (0.79–2.08) | 1.10 (1.02–1.17) |
| nnU-Net + DINOv3 + Clinical | External IEAH | Prior-shifted to development prevalence (20.5%) | 0.234 (0.207–0.261) | +1.18 (+1.03 to +1.33) | 1.32 (0.79–2.05) | 0.47 (0.41–0.53) |
| nnU-Net + DINOv3 + Clinical | External IEAH | Prior-shifted to external prevalence (37.6%) (illustration only) | 0.193 (0.166–0.221) | +0.33 (+0.18 to +0.47) | 1.32 (0.80–2.13) | 0.83 (0.76–0.91) |
| nnU-Net + DINOv3 | Development (OOF) | As published (class-balanced meta-learner) | 0.195 (0.180–0.212) | -1.32 (-1.41 to -1.23) | 1.26 (0.92–1.69) | 2.20 (2.12–2.28) |
| nnU-Net + DINOv3 | External IEAH | As published (class-balanced meta-learner) | 0.190 (0.164–0.216) | -0.16 (-0.30 to -0.03) | 1.41 (0.84–2.25) | 1.09 (1.02–1.16) |
| nnU-Net | Development (OOF) | As published (class-balanced meta-learner) | 0.218 (0.210–0.227) | -1.31 (-1.35 to -1.27) | 2.38 (1.70–3.14) | 2.33 (2.29–2.37) |
| nnU-Net | External IEAH | As published (class-balanced meta-learner) | 0.215 (0.202–0.228) | -0.34 (-0.40 to -0.28) | 2.93 (1.58–5.47) | 1.21 (1.17–1.25) |
| Clinical | Development (OOF) | As published (class-balanced meta-learner) | 0.175 (0.163–0.187) | +0.90 (+0.74 to +1.08) | 0.22 (0.08–0.39) | 0.55 (0.48–0.62) |
| Clinical | External IEAH | As published (class-balanced meta-learner) | 0.285 (0.262–0.309) | +1.77 (+1.53 to +2.06) | 0.29 (0.05–0.63) | 0.33 (0.28–0.39) |

*Calibration-in-the-large: logistic intercept with the logit of the prediction as offset (ideal 0); slope: ideal 1; E/O: mean predicted probability divided by observed prevalence. 2000 stratified bootstrap resamples. Prior shift: logit p + logit(prevalence), replacing the implied 50% prevalence of the class-balanced meta-learner; discrimination is unchanged. The external-prevalence shift is shown for illustration only (it uses information not available before external validation).*

**Supplementary Table S9. Operating points at the prespecified threshold and at post hoc sensitivity-oriented thresholds fixed on development data**

| **Model** | **Operating point** | **Threshold** | **Dev. sens / spec** | **External sensitivity (95% CI)** | **External specificity (95% CI)** | **External PPV (95% CI)** | **External NPV (95% CI)** | **External TP / FN / FP / TN** |
| --- | --- | --- | --- | --- | --- | --- | --- | --- |
| nnU-Net + DINOv3 + Clinical | Youden-J (prespecified) | 0.512 | 0.68 / 0.79 | 0.41 (0.28–0.57) | 0.91 (0.82–0.96) | 0.74 (0.54–0.87) | 0.72 (0.62–0.80) | 17 / 24 / 6 / 62 |
| nnU-Net + DINOv3 + Clinical | Development sensitivity ≥ 0.80 | 0.419 | 0.80 / 0.61 | 0.66 (0.51–0.78) | 0.71 (0.59–0.80) | 0.57 (0.43–0.70) | 0.77 (0.66–0.86) | 27 / 14 / 20 / 48 |
| nnU-Net + DINOv3 + Clinical | Development sensitivity ≥ 0.90 | 0.315 | 0.90 / 0.32 | 0.83 (0.69–0.91) | 0.49 (0.37–0.60) | 0.49 (0.38–0.61) | 0.82 (0.68–0.91) | 34 / 7 / 35 / 33 |
| nnU-Net + DINOv3 | Youden-J (prespecified) | 0.521 | 0.65 / 0.80 | 0.41 (0.28–0.57) | 0.94 (0.86–0.98) | 0.81 (0.60–0.92) | 0.73 (0.63–0.81) | 17 / 24 / 4 / 64 |
| nnU-Net + DINOv3 | Development sensitivity ≥ 0.80 | 0.409 | 0.80 / 0.61 | 0.68 (0.53–0.80) | 0.69 (0.57–0.79) | 0.57 (0.43–0.70) | 0.78 (0.66–0.87) | 28 / 13 / 21 / 47 |
| nnU-Net + DINOv3 | Development sensitivity ≥ 0.90 | 0.356 | 0.90 / 0.44 | 0.76 (0.61–0.86) | 0.59 (0.47–0.70) | 0.53 (0.40–0.65) | 0.80 (0.67–0.89) | 31 / 10 / 28 / 40 |
| nnU-Net | Youden-J (prespecified) | 0.447 | 0.69 / 0.80 | 0.44 (0.30–0.59) | 0.88 (0.78–0.94) | 0.69 (0.50–0.83) | 0.72 (0.62–0.81) | 18 / 23 / 8 / 60 |
| nnU-Net | Development sensitivity ≥ 0.80 | 0.431 | 0.80 / 0.34 | 0.51 (0.36–0.66) | 0.84 (0.73–0.91) | 0.66 (0.48–0.80) | 0.74 (0.63–0.83) | 21 / 20 / 11 / 57 |
| nnU-Net | Development sensitivity ≥ 0.90 | 0.429 | 0.90 / 0.17 | 0.54 (0.39–0.68) | 0.84 (0.73–0.91) | 0.67 (0.50–0.80) | 0.75 (0.64–0.83) | 22 / 19 / 11 / 57 |

*Thresholds were derived from development out-of-fold predictions (Youden-J prespecified; sensitivity ≥ 0.80 and ≥ 0.90 post hoc) and applied unchanged to the external cohort. Wilson 95% CIs.*

**Supplementary Table S10. Net benefit in the external cohort at threshold probabilities of 0.10–0.40**

| **Threshold** | **Treat all** | **nnU-Net + DINOv3 + Clinical** | **nnU-Net + DINOv3** | **nnU-Net** | **Clinical (TabPFN)** | **Ensemble minus treat-all** | **Ensemble minus nnU-Net** |
| --- | --- | --- | --- | --- | --- | --- | --- |
| 0.10 | 0.307 | 0.307 (0.215–0.419) | 0.307 (0.215–0.419) | 0.307 (0.215–0.419) | 0.214 (0.134–0.299) | -0.000 (-0.000 to +0.000) | +0.000 (+0.000 to +0.000) |
| 0.15 | 0.266 | 0.266 (0.169–0.385) | 0.266 (0.169–0.385) | 0.266 (0.169–0.385) | 0.154 (0.084–0.230) | +0.000 (-0.000 to +0.000) | +0.000 (+0.000 to +0.000) |
| 0.20 | 0.220 | 0.220 (0.117–0.337) | 0.222 (0.117–0.346) | 0.220 (0.117–0.346) | 0.094 (0.037–0.158) | +0.000 (-0.023 to +0.016) | +0.000 (-0.023 to +0.016) |
| 0.25 | 0.168 | 0.190 (0.080–0.312) | 0.196 (0.086–0.318) | 0.168 (0.058–0.303) | 0.055 (0.012–0.107) | +0.021 (-0.024 to +0.064) | +0.021 (-0.024 to +0.064) |
| 0.30 | 0.109 | 0.155 (0.046–0.274) | 0.155 (0.048–0.275) | 0.109 (-0.010–0.253) | 0.052 (0.008–0.106) | +0.046 (-0.018 to +0.102) | +0.046 (-0.018 to +0.102) |
| 0.35 | 0.040 | 0.145 (0.038–0.258) | 0.140 (0.032–0.255) | 0.040 (-0.087–0.195) | 0.045 (0.004–0.096) | +0.105 (+0.025 to +0.176) | +0.105 (+0.025 to +0.176) |
| 0.40 | -0.040 | 0.131 (0.024–0.242) | 0.125 (0.015–0.235) | -0.040 (-0.178–0.128) | 0.006 (-0.021–0.040) | +0.171 (+0.070 to +0.263) | +0.171 (+0.070 to +0.263) |

*Net benefit = TP/n − FP/n × pt/(1 − pt), with bootstrap 95% CIs (2000 resamples). Treat all is fixed by the external prevalence (37.6%). Below a threshold of 0.41, every external patient has an nnU-Net probability above the threshold, so nnU-Net equals treat-all.*

**Supplementary Table S11. Transportability of the six selected radiomic features: effect sizes, directional consistency, and distribution shift**

| **Feature** | **Direction** | **Development AUC (95% CI)** | **External AUC (95% CI)** | **Cliff’s δ dev / ext** | **Centers with same direction (of 5)** | **Center AUCs (BGCLR/BRSHH/GAZI/GEAH/IEAH)** | **SMD dev->ext, WHO 1 only** | **Center effect in WHO 1 (KW p, ε²)** |
| --- | --- | --- | --- | --- | --- | --- | --- | --- |
| Tumor NGTDM Strength (original) | lower in high grade | 0.773 (0.715–0.831) | 0.596 (0.486–0.706) | +0.55 / +0.19 | 5 | 0.74 / 0.70 / 0.85 / 0.78 / 0.60 | -0.15 | 7.7e-03, 0.03 |
| Tumor GLCM IMC2 (wavelet-HHL) | lower in high grade | 0.740 (0.679–0.801) | 0.564 (0.449–0.680) | +0.48 / +0.13 | 5 | 0.66 / 0.62 / 0.73 / 0.69 / 0.56 | +0.30 | 4.1e-07, 0.09 |
| Tumor GLCM IMC1 (wavelet-LHL) | higher in high grade | 0.768 (0.708–0.827) | 0.576 (0.462–0.689) | +0.54 / +0.15 | 5 | 0.73 / 0.70 / 0.79 / 0.64 / 0.58 | +0.09 | 1.2e-03, 0.04 |
| Peritumoral NGTDM Busyness (wavelet-LLL) | higher in high grade | 0.798 (0.740–0.857) | 0.642 (0.536–0.748) | +0.60 / +0.28 | 5 | 0.76 / 0.76 / 0.84 / 0.78 / 0.64 | -0.28 | 4.3e-04, 0.05 |
| Peritumoral NGTDM Strength (wavelet-LHL) | lower in high grade | 0.747 (0.689–0.805) | 0.670 (0.568–0.773) | +0.49 / +0.34 | 5 | 0.71 / 0.70 / 0.76 / 0.74 / 0.67 | +0.21 | 5.0e-04, 0.05 |
| Peritumoral GLCM IMC2 (wavelet-LLL) | lower in high grade | 0.761 (0.702–0.820) | 0.547 (0.432–0.661) | +0.52 / +0.09 | 5 | 0.74 / 0.66 / 0.76 / 0.81 / 0.55 | +0.16 | 5.7e-05, 0.06 |

*AUC in the development direction with DeLong 95% CI; Cliff’s δ = 2·AUC − 1. Centers with the same direction: centers with AUC > 0.5. SMD (external minus development) and Kruskal–Wallis test across the five centers (ε²) computed among WHO grade 1 tumors only.*

**Supplementary Table S12. Conventional logistic regression benchmarks and tumor volume**

| **Model** | **Development LOCO OOF AUC** | **External AUC (95% CI)** | **vs nnU-Net: ΔAUC (p)** | **vs final model: ΔAUC (p)** |
| --- | --- | --- | --- | --- |
| nnU-Net (reported model) | 0.726 | 0.732 (0.632–0.832) |  |  |
| Clinical model, TabPFN (reported model) | 0.615 | 0.645 (0.536–0.754) |  |  |
| nnU-Net + DINOv3 + Clinical (reported model) | 0.752 | 0.742 (0.643–0.842) |  |  |
| Logistic: age, sex, edema, bone invasion, location | 0.638 | 0.683 (0.575–0.792) | -0.048 (0.442) | -0.059 (0.223) |
| Logistic: the same five variables + log tumor volume | 0.676 | 0.697 (0.591–0.802) | -0.035 (0.533) | -0.046 (0.258) |
| Logistic: all seven clinical variables | 0.626 | 0.683 (0.577–0.789) | -0.049 (0.430) | -0.059 (0.202) |
| Logistic: log tumor volume | 0.647 | 0.625 (0.517–0.734) | -0.107 (0.061) | -0.117 (0.032) |
| Logistic: nnU-Net probability + log tumor volume | 0.720 | 0.728 (0.628–0.828) | -0.004 (0.907) | -0.014 (0.655) |
| Stack: nnU-Net + DINOv3 + logistic clinical model | 0.744 | 0.752 (0.652–0.851) | +0.020 (0.548) | +0.009 (0.143) |

*Unpenalized logistic regression (median imputation and scaling fitted within each training fold; no tuning). Development AUC: LOCO out-of-fold; external: model fitted on all development patients. ΔAUC = benchmark minus comparator with paired DeLong p. Tumor volume: manual tumor segmentation (log mL). Last row: the stack with the logistic clinical model in place of TabPFN (meta-learner tuned by LOCO).*

**Supplementary Table S13. Odds ratios of the logistic model with clinical variables and tumor volume (development cohort)**

| **Variable** | **Odds ratio (95% CI)** | **p** |
| --- | --- | --- |
| Age (per SD) | 1.16 (0.87-1.55) | 0.314 |
| Male sex | 1.39 (0.76-2.54) | 0.288 |
| Peritumoral edema | 2.03 (0.99-4.16) | 0.053 |
| Bone invasion | 2.12 (1.07-4.20) | 0.032 |
| Location: parasagittal/falx | 0.77 (0.37-1.57) | 0.465 |
| Location: skull base | 0.46 (0.22-0.95) | 0.035 |
| Location: posterior fossa | 0.11 (0.01-0.84) | 0.033 |
| Location: other | 0.00 (0.00-inf) | 1.000 |
| Log tumor volume (per SD) | 2.01 (1.39-2.90) | <0.001 |

*Fitted on all development patients; reference location = convexity. The category “other” contains no high-grade tumors (quasi-complete separation), so its estimate is not interpretable.*

**Supplementary Methods S1. Patient-level aggregation of the nnU-Net output**

Within the manual tumor segmentation, six statistics of the voxel-wise high-grade probability were computed (mean, minimum, maximum, standard deviation, and first and third quartiles). They were standardized and combined into a patient-level probability by an L2-regularized logistic regression with class-balanced weights (scikit-learn 1.6.1). Its inverse regularization strength C was tuned with Optuna (35 trials, TPE sampler, seed 42) to maximize the mean LOCO validation AUC (selected C = 0.0020). Development probabilities are the LOCO out-of-fold predictions of this classifier; external probabilities come from the classifier refitted on all development patients and applied once. The standardized coefficients and the discrimination of each statistic used alone are shown in Supplementary Table S4.

**Supplementary Methods S2. nnU-Net training folds**

The nnU-Net input was the CE-T1-weighted volume cropped to the bounding box of the largest connected component of the manual tumor segmentation plus a 2-cm physical margin (zero-padded beyond the image border), without masking; the training target was the tumor segmentation encoded by grade group (label 1, WHO grade 1; label 2, WHO grade 2–3). The 3d_fullres configuration was trained with nnU-Net’s default five-fold cross-validation, i.e., random patient-level splits of the 346 development patients (Supplementary Table S5). For each development patient, the voxel-wise probability of the high-grade label was taken from the fold model that had not been trained on that patient (out-of-fold); for external patients, the probabilities of the five fold models were averaged. No external patient was part of any nnU-Net training or validation fold. Each fold was trained for up to 1000 epochs with early stopping on the exponential moving average of the mean foreground pseudo-Dice, with the network’s default resampling to 2×1×1 mm.

The two fold structures are not nested: the nnU-Net probability map of a development patient was produced by a fold model trained on patients from all four centers, including the patient’s own center, so the development estimates of the nnU-Net classifier, and of stacks containing it, are out-of-fold at the patient level but not at the center level. For all LOCO-tuned learners, hyperparameters were selected on the mean LOCO validation AUC and the out-of-fold predictions were produced with the same folds, which can also make development estimates optimistic. External patients were not used for training, tuning, feature selection, or threshold selection.

**Supplementary Methods S3. Radiomics feature extraction, selection, and classification**

Features were extracted with PyRadiomics 3.1.0 from the CE-T1-weighted images, using the tumor segmentation and the manually edited 5-mm peritumoral shell as regions of interest. Preprocessing, which applied to the radiomics pipeline only, consisted of resampling to 1×1×1-mm voxels (B-spline interpolation for images, nearest neighbor for masks), z-score normalization of the image (normalizeScale 100, voxelArrayShift 300) with removal of intensities beyond ±3 SD, and fixed-bin-width discretization with a bin width of 5 on the normalized scale (padDistance 10, minimum ROI dimensionality 2, minimum ROI size 8 voxels, mask correction enabled). Image types were the original image, Laplacian-of-Gaussian filtered images (σ = 2, 3, 4, and 5 mm), and all eight wavelet decompositions; feature classes were first order, GLCM, GLRLM, GLSZM, GLDM, and NGTDM for both regions and shape for the tumor. This yielded 1,223 tumor features (107 original, including 14 shape features; 372 Laplacian-of-Gaussian; 744 wavelet) and 1,209 peritumoral features.

Feature selection used the development cohort only. (i) Reproducibility: the intraclass correlation coefficient ICC(2,1) was computed between features derived from two readers’ segmentations of 23 development cases; tumor features with ICC > 0.80 were retained (1,120 of 1,223), and the peritumoral set was restricted to the same feature definitions, giving 2,234 candidate features. (ii) Within each of five stratified folds of the pooled development cohort, using the training portion only, features were median-imputed and z-scored, ranked by univariate ROC AUC, and filtered for redundancy (|Pearson r| > 0.90, retaining the feature with the higher AUC); the 20 highest-ranked features were passed to forward sequential feature selection with a TabPFN classifier (objective ROC AUC, two-fold internal cross-validation, eight features per fold). (iii) Features selected in at least three of the five folds were retained (six features; Supplementary Table S2). Because these folds were stratified random splits of the pooled development cohort rather than the LOCO folds used for evaluation, the development out-of-fold AUC of the radiomics model may be optimistic; the external evaluation is unaffected.

Classification used TabPFN (tabpfn 8.0.7, version-3 classifier weights). The number of ensemble members, softmax temperature, averaging before or after the softmax, and probability balancing were tuned with Optuna (100 trials, TPE sampler, seed 42) to maximize the mean LOCO validation AUC. The selected configurations were 16 ensemble members, softmax temperature 0.79, averaging before the softmax, and no probability balancing for the radiomics model, and 8 ensemble members, softmax temperature 0.50, averaging after the softmax, and no probability balancing for the clinical model. Median imputation and z-score scaling were fitted within each training fold, and the final models were refitted on all development patients.

**Supplementary Methods S4. Explainability**

The meta-learner is linear in the standardized base-model probabilities, logit P = b0 + Σk wk(pk − μk)/σk for k ∈ {nnU-Net, DINOv3, clinical}, so each prediction decomposes exactly into additive contributions of the base models. The clinical term a(pc − μc), with a = wc/σc, was divided among the clinical variables using exact interventional SHAP values φj of the clinical TabPFN model with the development-mean patient as reference (all 2^11 coalitions of the 11 encoded inputs, grouped into seven variables). Because pc(x) − μc = Σj φj + (f(reference) − μc), each variable receives aφj and the constant a(f(reference) − μc) is added to the base value (−0.29). The submitted version instead multiplied φj by (pc − μc)/Σj φj, which is unstable when Σj φj is close to zero; the model-level contributions of nnU-Net and DINOv3 are unaffected. TabPFN outputs were computed in batches of at most 512 patients, because its predictions changed slightly for larger batches; the residual difference from the stored probabilities (at most 0.001 on the log-odds scale) was distributed over the variables in proportion to |φj|. For Supplementary Figure S5, the patient with the median predicted probability in each cell of the external confusion matrix at the prespecified threshold was selected.
